# Supplementary material for: Analysis of Endangered Andalusian Black Cattle (Negra Andaluza) Reveals Genetic Reservoir for Bovine Black Trunk
Source: Animals (Basel). 2024 Apr 8;14(7):1131. doi: 10.3390/ani14071131 (PMC11010997; doi:10.3390/ani14071131)

Figure S2. Andalusian Black Cattle Breed process of configuration, development and consolidation.

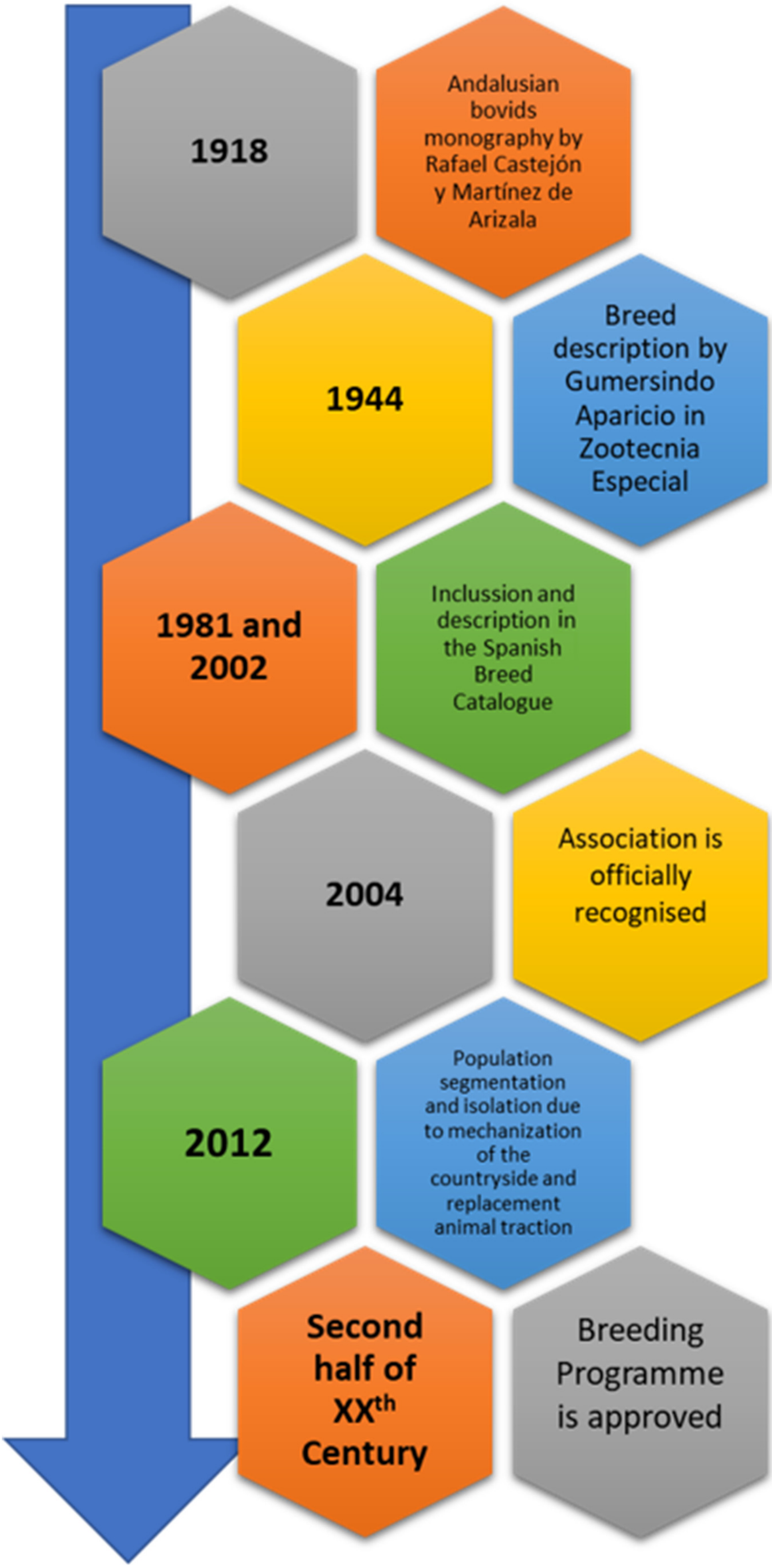

Supplement: Supplementary file 1 [file animals-14-01131-s001.zip › Figure S2.pdf]
