# Supplementary material for: Analysis of Endangered Andalusian Black Cattle (Negra Andaluza) Reveals Genetic Reservoir for Bovine Black Trunk
Source: Animals (Basel). 2024 Apr 8;14(7):1131. doi: 10.3390/ani14071131 (PMC11010997; doi:10.3390/ani14071131)
Supplement: Supplementary file 1 [file animals-14-01131-s001.zip › Supplementary Table S1.pdf]

Supplementary Table S1. Black Andalusian cattle breed data fact sheet.

**Breed standard**	The breed exhibits a subconcave profile, average proportions and lengths, and significant sexual dimorphism. They possess great rusticity and good maternal aptitude, with a production of one calf per year. The height at the withers is 140 cm in males and 135 cm in females. The weight for females ranges from 550 to 600 kg, and for males, it falls between 850 and 900 kg.
**Criteria for herdbook inscription**	The breed rating system for herdbook inscription is based on a morphological rating of the animal in seven body areas, depending on the degree of similarity to the breed standard. The rating is conducted on animals with sufficient body development and at least 24 months of age. Each area is scored on a scale of 1 to 10 points. Any area scored below 5 results in the disqualification of the animal. Two results are established: Non-eligible and Eligible, further categorized into four subcategories based on the sum of ratings of the body regions.<br>• Sufficient: 70 to 73 points for males, 65 to 69 points for females<br>• Good: 74 to 79 points for males, 70 to 75 points for females<br>• Very Good: 80 to 89 points for males, 76 to 86 points for females<br>• Excellent: ≥90 points for males, ≥87 points for females
**Extension**	Perfectly adapted to extensive systems with stational supplementation.
**Herds size**	The average herd size is 96.409 animals. The total population is 1797 animals distributed across 324 farms.
**Orientation**	This breed is oriented towards meat production, usually in close relation to extensive farms in the Andalusian Community. Females are characterized by high fertility and longevity under challenging conditions due to their rusticity and adaptability.
**Husbandry and health practices**	The breed's rusticity allows for extensive rearing, utilizing resources from dehesas and supplemented during scarcity. This type of livestock can utilize stubble and grains from the grassy areas where they are raised.
**Reproductive management**	The reproductive management relies on a natural mating system in freedom, with no artificial insemination. The age of puberty for females is reached at 18-20 months. The breed achieves 80% fecundity, 95% fertility, and a 1.15% rate of twin births.
**Main products**	The most representative type of calves weighs around 190-220 kg (live weight) at weaning (6-7 months) until they reach 14 months of age. Calves, whether pure or industrially crossed, have good potential for organic meat production with appropriate maturation and recognized quality in the market.
**Conservation official recognition**	Threatened.
**Conservation structures status**	Although the authorization for managing the breed's herdbook was granted to the Association of Breeders of the Black Andalusian Cattle Breed in 2005, its breeding program was officially approved seven years later, in December 2012.

Access the Resolution of April 28, 2020, from the Spanish General Directorate of Livestock approving the breeding program of the Black Andalusian Cattle Breed here. Additional information can be found on the ARCA platform of the Spanish Ministry of Agriculture, Fisheries, and Food here.
