# Supplementary material for: Analysis of Endangered Andalusian Black Cattle (Negra Andaluza) Reveals Genetic Reservoir for Bovine Black Trunk
Source: Animals (Basel). 2024 Apr 8;14(7):1131. doi: 10.3390/ani14071131 (PMC11010997; doi:10.3390/ani14071131)
Supplement: Supplementary file 1 [file animals-14-01131-s001.zip › Supplementary Table S2.pdf]

Supplementary Table S2. Descriptive statistics of average age (years) of the parents at the birth of their offspring and generation intervals or generation length (years) for the four gametic routes in the Black Andalusian cattle.

Average age (years) of the parents at the birth of their offspring						
Parameter / Population set	Mean age of the parents at the birth of their offspring	Sire to son	Damr to son	Sire to daughter	Dam to daughter	Total
Historical (n=8555)	N	3448	3559	3929	4058	14994
	Mean	6.18	5.81	6.15	5.74	5.97
	SD	2.86	3.09	2.90	3.08	2.99
	SEM	0.049	0.052	0.046	0.049	0.024
Current (n=2472)	N	565	591	1835	1877	4868
	Mean	5.95	6.71	5.93	5.96	6.04
	SD	2.47	3.19	2.76	3.04	2.90
	SEM	0.104	0.134	0.116	0.128	0.042
Generation intervals, generation length or average age (years) of the parents at birth of their offspring that in their turn will produce the next generation of breeding animals						
Parameter / Population set	Gametic route	Sire to son	Damr to son	Sire to daughter	Dam to daughter	Total
Historical (n=8555)	N	62	72	1484	1552	3170
	Mean	5.91	5.06	5.95	5.30	5.61
	SD	2.98	3.04	2.91	2.84	2.90
	SEM	0.378	0.359	0.076	0.072	0.052
Current (n=2472)	N	24	25	990	1026	2065
	Mean	5.71	6.25	5.86	5.49	5.68
	SD	3.04	3.13	2.85	2.85	2.87
	SEM	0.621	0.659	0.584	0.583	0.063
